# Effects of extremely low-frequency sinusoidal electromagnetic field therapy on survival and vascularization in a rat random-pattern skin flap model

**DOI:** 10.1016/j.jpra.2026.05.040

**Published:** 2026-05-30

**Authors:** Mehmet Goktug Esmer, Aysun Ozbay Onal, Mustafa Onal, Sevda Demir, Seden Yurtoglu, Merdan Serin

**Affiliations:** a Istanbul Medeniyet University, Department of Plastic, Reconstructive and Aesthetic Surgery, Faculty of Medicine, Turkey; b Istanbul Aydın University, Department of Histology and Embryology, Faculty of Medicine, Turkey; c Yeditepe University, Faculty of Engineering and Natural Sciences, Department of Genetics and Bioengineering, Turkey; d Istanbul Maltepe University, Faculty of Medicine, Turkey

**Keywords:** Electromagnetic field, Random-pattern skin flap, Wound healing, Angiogenesis, Apoptosis

## Abstract

**Aim:**

This study aimed to evaluate the dose-dependent effects of extremely low-frequency sinusoidal electromagnetic fields (ELF-EMF) on survival and vascularization of random-pattern dorsal skin flaps in rats and to investigate associated histological and molecular changes.

**Materials and methods:**

Twenty-one male Sprague-Dawley rats underwent McFarlane dorsal random-pattern flap surgery and were randomly assigned to three groups (n = 7/group): control, low-dose ELF-EMF (0.2 mT), and high-dose ELF-EMF (5 mT). Sinusoidal ELF-EMF exposure (50 Hz) was administered twice daily for 15 min for 14 postoperative days. Flap viability was assessed by digital planimetry. Histological evaluation included hematoxylin-eosin and Masson's trichrome staining. TNF-α immunohistochemistry and quantitative real-time PCR analysis of apoptosis-, inflammation-, and angiogenesis-related genes were performed.

**Results:**

The low-dose ELF-EMF group demonstrated significantly higher flap viability than both the control and high-dose groups (76.3 ± 6.9% vs. 55.2 ± 9.1% and 55.7 ± 11.3%, respectively; p < 0.05). Histological analyses showed enhanced re-epithelialization, increased vascularization, improved collagen organization, and reduced inflammatory cell infiltration in the low-dose group. High-dose ELF-EMF did not improve flap survival and was associated with suppression of VEGF and MAPK pathway-related gene expression. The discordance between increased TNF-α mRNA expression and reduced immunohistochemical staining in the low-dose group suggests temporally dynamic and post-transcriptional regulation.

**Conclusion:**

Low-dose sinusoidal ELF-EMF improved survival and histological markers of repair in a rat random-pattern skin flap model, whereas high-dose exposure did not confer benefit. These findings support the existence of a dose-dependent biological window for ELF-EMF therapy. Given the exploratory preclinical nature of the study, further mechanistic and translational investigations are required before clinical application.

## Introduction

Random-pattern skin flaps are fundamental reconstructive techniques widely used to repair soft-tissue defects throughout the body. Their principal advantage lies in technical simplicity and versatility; however, flap survival is often limited by inadequate perfusion in the distal portion of the flap, resulting in partial necrosis, delayed wound healing, and compromised functional and aesthetic outcomes.^1^^,^^7^^,^^8^ Despite refinements in flap design and perioperative management, distal ischemia remains a significant challenge in reconstructive surgery.

Numerous experimental and clinical strategies have been investigated to improve flap viability, including surgical delay, ischemic preconditioning, vasodilator therapy, antioxidant agents, hyperbaric oxygen therapy, stem cell-based treatments, and growth factor administration.^1^^,^^18^ Although many of these approaches have shown promising results, their clinical adoption may be constrained by cost, invasiveness, technical complexity, or inconsistent efficacy. Accordingly, there is continued interest in identifying noninvasive and cost-effective adjunctive therapies that can reliably enhance flap survival.

Extremely low-frequency electromagnetic fields (ELF-EMFs) have emerged as a potential therapeutic modality with applications in orthopedics, rehabilitation, and tissue repair.^2^, ^3^, ^4^, ^5^, ^6^ ELF-EMF exposure has been reported to stimulate fibroblast proliferation, increase collagen synthesis, enhance angiogenesis, modulate inflammatory responses, and accelerate epithelial regeneration.^2^^,^^3^^,^^11^, ^12^, ^13^ In both in vitro and in vivo models, electromagnetic stimulation has been shown to influence key signaling pathways involved in wound healing, including mitogen-activated protein kinase (MAPK), nuclear factor-kappa B (NF-κB), and apoptosis-related pathways.^21^, ^22^, ^23^, ^24^, ^25^, ^26^, ^27^, ^28^, ^29^, ^30^, ^31^, ^32^, ^33^, ^34^, ^35^, ^36^, ^37^

Despite these encouraging findings, the biological effects of ELF-EMF are highly dependent on exposure parameters such as frequency, waveform, intensity, and treatment duration. Experimental studies have suggested that electromagnetic stimulation exhibits a non-linear dose-response relationship, often described as a “biological window,” in which only specific combinations of field strength and frequency produce beneficial effects, whereas exposures outside this range may be ineffective or even detrimental.^5^^,^^6^^,^^35^ Defining this therapeutic window is essential for optimizing the clinical utility of ELF-EMF therapy.

Several studies have demonstrated beneficial effects of pulsed or sinusoidal electromagnetic fields on wound healing and flap survival.^9^^,^^10^^,^^15^ Proposed mechanisms include improved endothelial function, stimulation of angiogenic mediators such as vascular endothelial growth factor (VEGF), modulation of inflammatory cytokines, and regulation of oxidative stress and apoptosis.^16^, ^17^, ^18^, ^19^, ^20^, ^21^, ^22^, ^23^, ^24^, ^25^, ^26^, ^27^, ^28^, ^29^, ^30^ However, published results remain heterogeneous, and the molecular mechanisms underlying dose-dependent responses are not fully understood.

In particular, relatively few studies have examined sinusoidal ELF-EMF exposure in standardized random-pattern skin flap models using integrated macroscopic, histological, immunohistochemical, and molecular analyses. Furthermore, the differential effects of low- versus high-intensity ELF-EMF on regenerative signaling pathways have not been comprehensively characterized.

Therefore, the aim of this study was to evaluate the effects of low-intensity (0.2 mT) and high-intensity (5 mT) sinusoidal ELF-EMF exposure at 50 Hz on survival and histological markers of vascularization in McFarlane dorsal random-pattern skin flaps in rats. Because direct perfusion measurements such as laser Doppler flowmetry were not performed, vascular responses were assessed indirectly through flap survival, histological vascularity, and angiogenesis-related molecular markers. By integrating macroscopic, histological, immunohistochemical, and gene-expression analyses, we sought to characterize dose-dependent biological responses and provide foundational preclinical data to guide future translational development of ELF-EMF as a noninvasive adjunctive therapy in reconstructive surgery.

## Materials and methods

### Study design and animals

This experimental study was conducted at the Maltepe University Experimental Animal Research Center following approval by the Institutional Animal Care and Use Committee (Protocol No 2025.02.02; approval date: February 27, 2025). All procedures were performed in accordance with institutional guidelines for the care and use of laboratory animals.

The study was designed as an exploratory preclinical investigation to evaluate the effects of low- and high-dose sinusoidal extremely low-frequency electromagnetic field (ELF-EMF) exposure on survival and histological markers of repair in a rat random-pattern skin flap model.

Twenty-one male Sprague-Dawley rats aged 6 weeks and weighing 250–300 g were included. Animals were randomly assigned to one of three groups (n = 7 per group):

1. Control group: No ELF-EMF exposure
2. Low-dose ELF-EMF group: 0.2 mT, 50 Hz
3. High-dose ELF-EMF group: 5 mT, 50 Hz

No formal a priori power analysis was performed. The sample size was selected based on previous studies employing similar random-pattern skin flap models and was considered appropriate for this exploratory proof-of-concept investigation. The primary outcome measure was percentage of viable flap area. Secondary outcomes included histological scores, immunohistochemical findings, and gene-expression profiles.

All animals were acclimatized for 7 days before the experiment and housed under standardized environmental conditions (22 ± 2 °C, 50 ± 5% humidity, 12-hour light/dark cycle) with ad libitum access to food and water.

### Surgical procedure

All rats underwent dorsal random-pattern skin flap elevation using the McFarlane flap model.

Following induction of general anesthesia, the dorsal region was shaved and disinfected with povidone-iodine solution. A caudally based random-pattern skin flap measuring 9 × 3 cm was designed on the dorsum. The flap was elevated including the panniculus carnosus and immediately repositioned to its original bed. The flap was then secured using interrupted 4–0 polypropylene sutures.

Flap dimensions and operative technique were standardized in all animals.

### Electromagnetic field exposure

ELF-EMF exposure was delivered using a custom-built cylindrical solenoid coil with the following characteristics:

• Inner diameter: 15 cm
• Number of turns: 400 copper wire turns
• Waveform: sinusoidal
• Frequency: 50 Hz

The solenoid was connected to a waveform generator and power amplifier capable of producing stable sinusoidal electromagnetic fields.

Animals were positioned centrally within the coil such that the dorsal flap area was located within the region of maximal field uniformity. The low-dose group received a magnetic flux density of 0.2 mT (RMS), whereas the high-dose group received 5 mT (RMS).

Each exposure session lasted 15 min and was administered twice daily for 14 consecutive postoperative days.

Field intensity was verified before each session using a calibrated gaussmeter.

Control animals underwent identical restraint and handling procedures within the inactive exposure apparatus to control for environmental and handling-related effects.

Although comprehensive three-dimensional dosimetric mapping was not performed, all animals were positioned consistently within the same central exposure zone to minimize variability in field exposure.

### Macroscopic assessment of flap viability

Flap viability was assessed on postoperative day 7.

Standardized digital photographs were obtained under identical lighting and camera conditions. Viable and necrotic regions were quantified using ImageJ software (National Institutes of Health, Bethesda, MD, USA).

The percentage of viable flap area was calculated as:

$$\text{Flap}\;\text{viability}\;(\%)=\frac{\text{Viable}\;\text{area}}{\text{Total}\;\text{flap}\;\text{area}}\times \;100$$

Measurements were performed independently by two investigators blinded to treatment allocation.

### Histological analysis

Following euthanasia, tissue samples were harvested from the distal flap region and fixed in 10% neutral-buffered formalin for 24 h. Samples were dehydrated through graded alcohols, cleared in xylene, and embedded in paraffin.

Serial 5-µm sections were prepared and stained with:

• Hematoxylin-eosin (H&E) for general tissue architecture
• Masson’s trichrome for collagen deposition and extracellular matrix organization

Histological evaluation included assessment of:

• Re-epithelialization
• Inflammatory cell infiltration
• Vascularization
• Collagen deposition

Each parameter was scored using a semi-quantitative scale from 0 to 3:

• 0 = absent/minimal
• 1 = mild
• 2 = moderate
• 3 = marked

All slides were evaluated by a blinded histologist.

Because scoring was performed by a single observer, interobserver variability was not assessed.

### Immunohistochemistry

Paraffin-embedded sections (5 µm) were deparaffinized in xylene and rehydrated through graded alcohols.

Antigen retrieval was performed using citrate buffer (pH 6.0). Endogenous peroxidase activity was blocked with 3% hydrogen peroxide.

Sections were incubated overnight at 4 °C with rabbit polyclonal anti-TNF-α antibody (Affinity Biosciences, catalog no AF7014; dilution 1:100).

After washing, sections were incubated with HRP-conjugated secondary antibody. Immunoreactivity was visualized using 3,3′-diaminobenzidine (DAB) and counterstained with hematoxylin.

TNF-α staining intensity in keratinocytes, fibroblasts, endothelial cells, and inflammatory cells was assessed semi-quantitatively by a blinded observer.

### RNA isolation and quantitative real-time PCR

Total RNA was extracted from flap tissue using the Direct-zol™ RNA MiniPrep Plus Kit (Zymo Research, Irvine, CA, USA; catalog no R2072) according to the manufacturer’s instructions.

RNA concentration and purity were determined spectrophotometrically.

Complementary DNA (cDNA) was synthesized from 200 ng of total RNA using a commercial reverse transcription kit.

Quantitative real-time PCR (qRT-PCR) was performed using SYBR Green master mix under standard cycling conditions.

Target genes included:

• Apoptosis-related genes: BAX, BCL2, CASP8, CASP9, CYTO.C
• Inflammatory marker: TNF-α
• Signaling pathway genes: MAPK1, MAPK3, MAPK14
• Angiogenesis marker: VEGF

GAPDH was used as the internal reference gene.

All reactions were performed in triplicate, and no-template controls were included to exclude contamination.

Relative gene expression was calculated using the 2^-ΔΔCt^ method.

Primer sequences are provided in Table 1.

**Table 1:** Primer sequences used for qRT-PCR analysis.

Primer ID	Sequence (5´−3´ )	Primer ID	Sequence (5´−3´ )
CASP8_F	CCAGAGACTCCAGGAAAAGAGA	CASP8_R	CGGATGTCCCAGTACGAGATAG
CASP9_F	AGTTCCCGGGTGCTGTCTA	CASP9_R	ACTCGTCTTTCTGGTACCG
BAX_F	TTGCTTCAGGGTTTCATCCA	BAX_R	GTTCTTCGACTCGCTCACAGA
BCL-2_F	TGGCCAAGGGTCAGAGTTAAA	BCL-2_R	ATGAGGCGTTCTTCTCTCCGGT
CYTO.C_F	GAGGCAAGCATAAGACTGGA	CYTO.C_R	CTCCTATGGGACTACCTCAT
TNF-α_F	TGAGACCTTCAACACCCCAG	TNF-α_R	TTCATGAGGTAGTCTGTCAGGTCC
MAPK1_F	ATGGCGGCGGCGGCGGCGGCGG	MAPK1_R	TCAGGCGGCGGCGGCGGCGGCG
MAPK3_F	AGCTGACCTGGTGTTGATGA	MAPK3_R	TCTGCTGCTGCTGATGATGT
MAPK14_F	GCTGACCTGGTGTTGATGA	MAPK14_R	TCTGCTGCTGCTGATGATGT
VEGF_F	GAGGGCAGAATCATCACGAAGT	VEGF_R	TCTCGATTGGATGGCAGTAGCT
GAPDH_F	TTGTCTCCTGCGACTTCAACAG	GAPDH_R	GAGTGTTAAAGGTAGGGTCTGG

### Statistical analysis

Statistical analyses were performed using GraphPad Prism version 10.0.2 (GraphPad Software, San Diego, CA, USA).

Normality of continuous variables was assessed using the Shapiro-Wilk test.

• Normally distributed variables were analyzed using one-way analysis of variance (ANOVA) followed by Tukey’s multiple-comparison test.
• Non-normally distributed variables and ordinal histological scores were analyzed using the Kruskal-Wallis test followed by Dunn’s multiple-comparison test.

Gene-expression analyses were performed using ΔCt-derived values, with results presented as relative expression levels calculated by the 2^-ΔΔCt^ method.

Data are reported as mean ± standard deviation (SD) or median (interquartile range [IQR]), as appropriate.

A two-sided p value < 0.05 was considered statistically significant.

## Results

A total of 21 rats completed the study, with seven animals in each group (control, low-dose ELF-EMF, and high-dose ELF-EMF). All animals tolerated the surgical procedure and the assigned exposure protocol without perioperative mortality or protocol deviations.

### Flap survival analysis

There was no statistically significant difference in total flap area among the three groups (p = 0.140), confirming that flap dimensions were comparable and that differences in viability were unlikely to be attributable to variations in surgical technique.

**Fig. 1:**
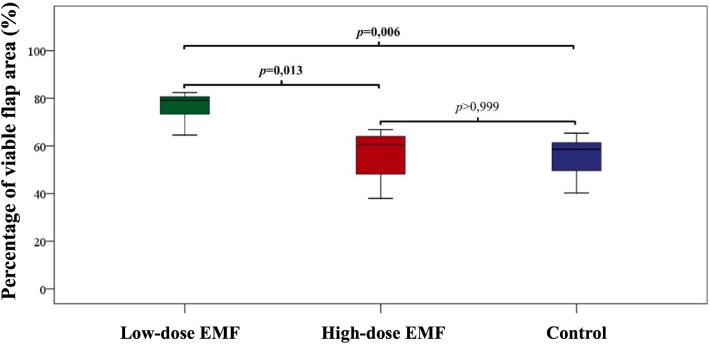
Percentage of viable flap area on postoperative day 7. Individual box plots show median, interquartile range, and full range. Low-dose ELF-EMF significantly increased flap survival compared with both control and high-dose groups.

The percentage of viable flap area differed significantly among groups (p = 0.003). The low-dose ELF-EMF group demonstrated the highest flap survival rate, with a mean viable area of 76.30 ± 6.88% (median 79.14%; interquartile range [IQR], 68.78–82.01). This was significantly greater than both the control group (55.16 ± 9.11%; median 58.59%; IQR, 46.35–62.14; p = 0.006) and the high-dose ELF-EMF group (55.65 ± 11.27%; median 60.42%; IQR, 43.22–64.72; p = 0.013). No significant difference was observed between the control and high-dose groups (p > 0.999) (Table 2, Fig. 1).

**Table 2:** Comparison and pairwise analyses of flap parameters among experimental groups.

Category	Variable	Low-dose EMF group (n = 7)	High-dose EMF group (n = 7)	Control group (n = 7)	p-value
Flap area	Total flap area (cm²)	30.64 ± 7.47 / 29.96 (24.18–40.15)	26.19 ± 7.13 / 26.31 (23.01–28.40)	23.72 ± 3.74 / 22.33 (21.85–25.91)	0.140
Flap viability	Viable flap area (%)	76.30 ± 6.88 / 79.14 (68.78–82.01)	55.65 ± 11.27 / 60.42 (43.22–64.72)	55.16 ± 9.11 / 58.59 (46.35–62.14)	**0.003**
Post-hoc comparisons					
Flap viability	Viable flap area (%)	76.30 ± 6.88	55.65 ± 11.27	-	**0.013**
		76.30 ± 6.88	-	55.16 ± 9.11	**0.006**
		-	55.65 ± 11.27	55.16 ± 9.11	>0.999

Data are presented as mean ± standard deviation and median (interquartile range, 25th–75th percentile). Group comparisons were performed using the Kruskal–Wallis test. Post-hoc p-values were obtained from pairwise comparisons following the Kruskal–Wallis test. A p-value < 0.05 was considered statistically significant. EMF: electromagnetic field.

These findings indicate that low-dose sinusoidal ELF-EMF significantly enhanced flap viability, whereas high-dose exposure did not provide measurable benefit.

### Histopathological scoring

Semi-quantitative histopathological analysis demonstrated significant differences among groups in all evaluated parameters.

**Re-epithelialization:** The low-dose ELF-EMF group exhibited significantly greater re-epithelialization compared with the control group (p = 0.0013), reflecting accelerated epidermal regeneration.

**Inflammatory Cell Infiltration:** Inflammation scores were significantly lower in the low-dose ELF-EMF group than in both the control and high-dose groups (p = 0.0063 for both comparisons), indicating attenuation of the inflammatory response.

**Vascularization:** Histological vascularization scores were significantly higher in the low-dose ELF-EMF group than in the control group (p = 0.0020) and the high-dose ELF-EMF group (p = 0.0100), suggesting enhanced neovascularization.

**Collagen Deposition:** Collagen organization and deposition were significantly increased in the low-dose ELF-EMF group compared with both the control (p = 0.0003) and high-dose groups (p = 0.0102), consistent with improved extracellular matrix remodeling.

**Fig. 2:**
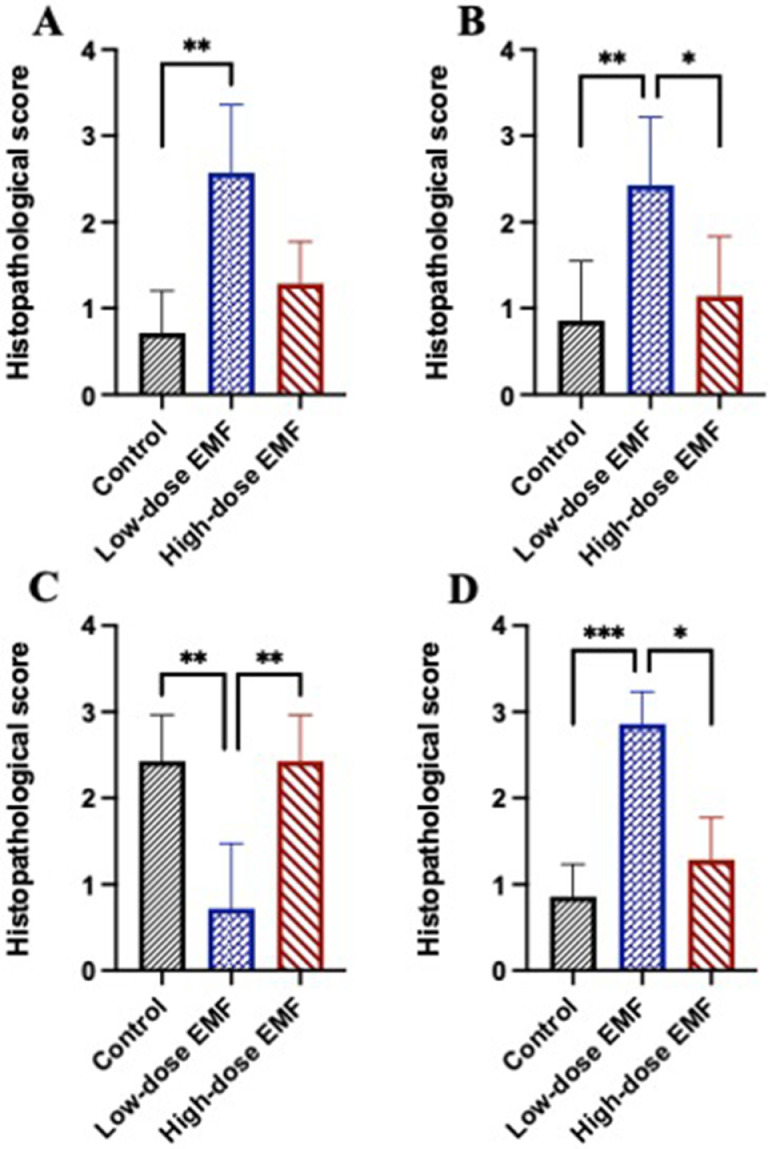
Semi-quantitative histological scores (0–3) for re-epithelialization, inflammation, vascularization, and collagen deposition.

Collectively, these findings demonstrate that low-dose ELF-EMF favorably influenced multiple histological markers of wound repair (Fig. 2).

### Histological observations

Representative hematoxylin-eosin and Masson's trichrome sections supported the quantitative scoring results.

In the low-dose ELF-EMF group, tissue sections demonstrated preserved epidermal continuity, reduced inflammatory infiltrate, increased vascular structures, and well-organized collagen bundles.

In the control group, epithelial disruption, edema, and dense inflammatory cell infiltration were evident.

**Fig. 3:**
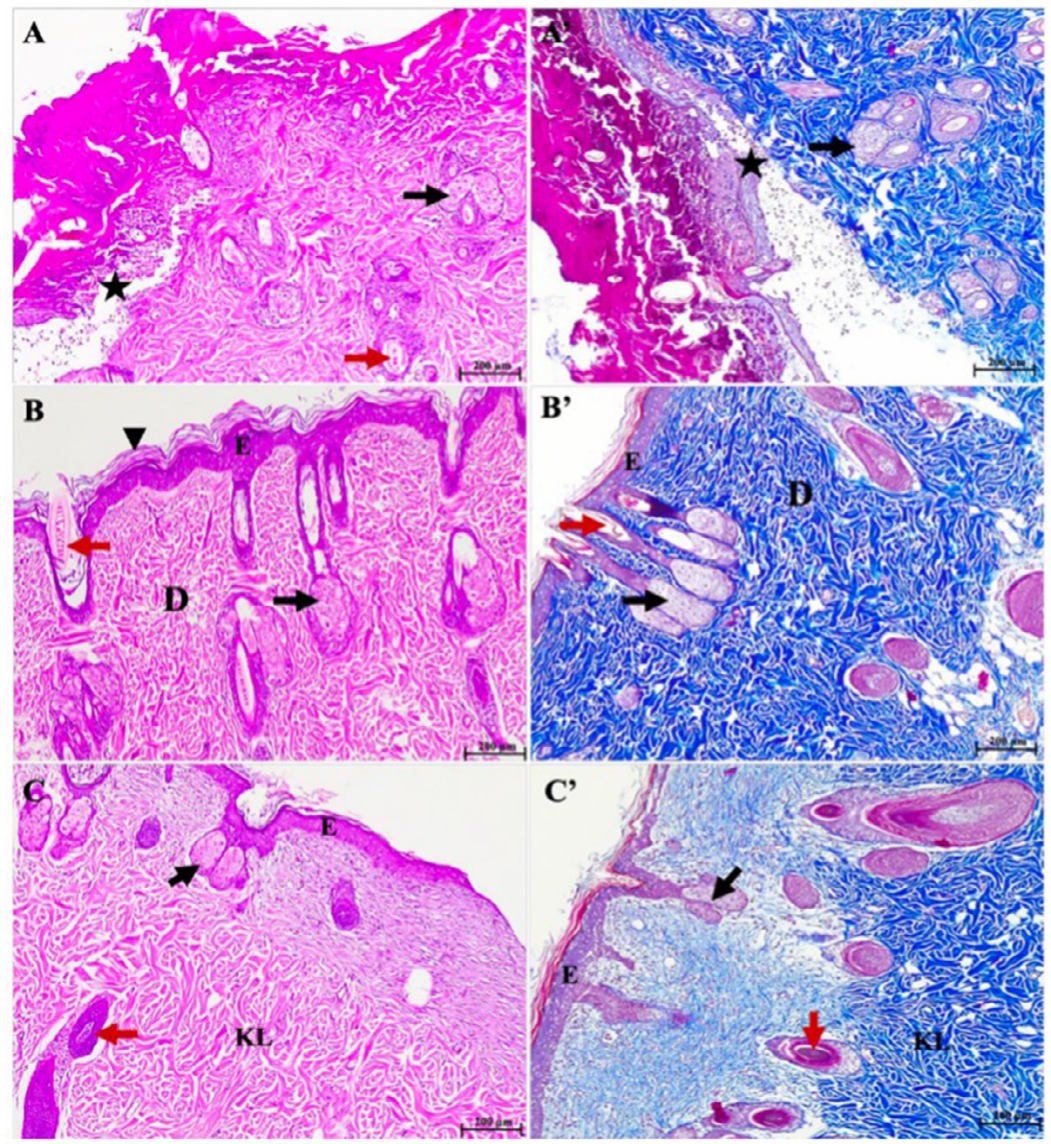
Representative histological images stained with hematoxylin-eosin and Masson's trichrome. E, epidermis; D, dermis; KL, collagen layer. Black arrows indicate vascular structures; red arrows indicate hair follicles; arrowheads indicate inflammatory cell infiltrates.

In the high-dose ELF-EMF group, epithelial thinning, vascular congestion, and less organized collagen architecture were observed, without the reparative features seen in the low-dose group (Fig. 3).

### TNF-α immunohistochemistry

TNF-α immunoreactivity differed qualitatively among groups.

The control and high-dose ELF-EMF groups exhibited stronger TNF-α staining in keratinocytes, fibroblasts, endothelial cells, and inflammatory cells, consistent with persistent inflammatory activity.

**Fig. 4:**
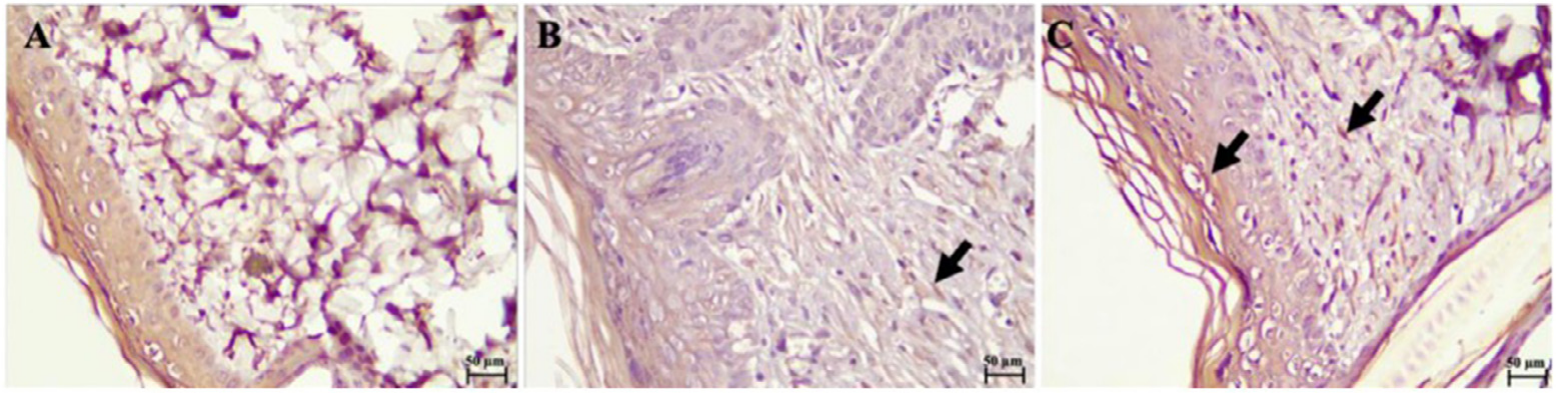
Immunohistochemical localization of TNF-α in skin flaps of experimental groups. Representative sections showing TNF-α expression in the control (A), low-dose EMF (B), and high-dose EMF (C) groups. Black arrows indicate TNF-α immunoreactivity in keratinocytes, fibroblasts, and inflammatory cells. Stronger cytoplasmic immunoreactivity was observed in the control and high-dose EMF groups compared with the low-dose EMF group. Scale bar: 50 μm; magnification × 40.

In contrast, the low-dose ELF-EMF group showed comparatively weak TNF-α immunoreactivity despite improved histological healing, suggesting a more controlled inflammatory response (Fig. 4).

### Quantitative real-time PCR analysis

**Fig. 5:**
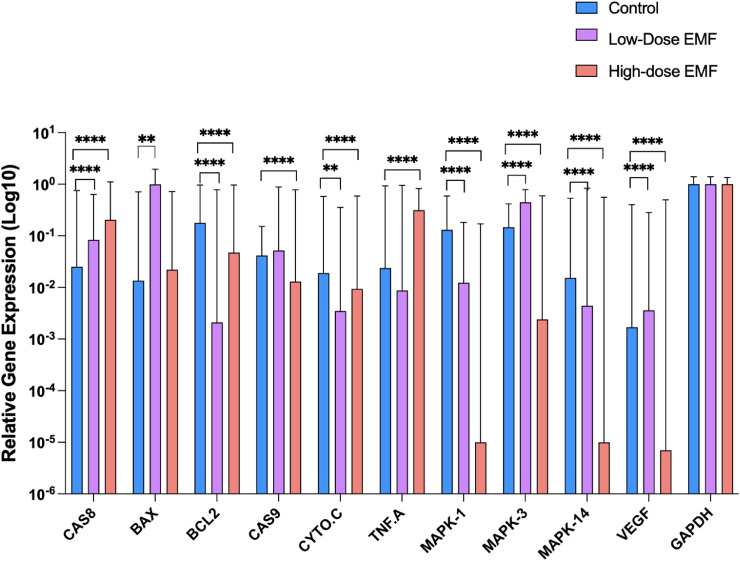
Relative mRNA expression levels determined by quantitative real-time PCR and normalized to GAPDH using the 2^-ΔΔCt^ method.

Gene-expression profiling revealed distinct dose-dependent molecular responses to ELF-EMF exposure (Fig. 5).

### Apoptosis-related genes

In the low-dose ELF-EMF group, apoptosis-related genes including CASP8, BAX, BCL2, and CASP9 were significantly upregulated compared with the control group. The most prominent increases were observed in BAX and BCL2 expression, suggesting activation of both stress-response and cytoprotective pathways.

In the high-dose ELF-EMF group, CASP8 remained elevated, whereas CASP9 expression was reduced relative to the low-dose group.

### Inflammation-related genes

TNF-α mRNA expression was significantly increased in the low-dose ELF-EMF group and remained elevated in the high-dose group.

However, the low-dose group demonstrated relatively weak TNF-α protein immunoreactivity on histological examination. This apparent discrepancy may reflect temporal differences between transcriptional activation and protein accumulation, post-transcriptional regulation, or the semi-quantitative nature of immunohistochemistry. Accordingly, these findings should be interpreted cautiously and do not necessarily indicate sustained pro-inflammatory activity.

### Angiogenesis and signaling pathways

VEGF expression showed mild upregulation in the low-dose ELF-EMF group, consistent with enhanced angiogenic activity.

In contrast, VEGF expression was markedly reduced in the high-dose group.

Similarly, MAPK pathway-related genes (MAPK1, MAPK3, and MAPK14) were significantly downregulated in the high-dose ELF-EMF group (p < 0.0001 for all comparisons), suggesting suppression of pathways involved in proliferation, angiogenesis, and stress adaptation.

### Overall molecular interpretation

Taken together, the gene-expression data suggest that low-dose ELF-EMF induces a coordinated biological response involving both adaptive stress signaling and reparative pathways. Concurrent upregulation of pro-apoptotic and anti-apoptotic transcripts likely reflects tightly regulated cellular remodeling rather than a uniformly beneficial anti-apoptotic effect.

In contrast, high-dose ELF-EMF was associated with suppression of VEGF and MAPK-related genes, findings consistent with impaired regenerative signaling and the absence of functional benefit.

Overall, these molecular findings complement the macroscopic and histological results and support the existence of a dose-dependent biological window for ELF-EMF therapy.

## Discussion

This experimental study demonstrated that low-dose sinusoidal extremely low-frequency electromagnetic field (ELF-EMF) exposure (0.2 mT, 50 Hz) significantly improved survival of random-pattern skin flaps in rats, whereas high-dose exposure (5 mT, 50 Hz) did not confer a measurable benefit. Animals treated with low-dose ELF-EMF showed a significantly greater percentage of viable flap area, accompanied by enhanced re-epithelialization, increased histological vascularization, improved collagen organization, and reduced inflammatory cell infiltration. In contrast, high-dose ELF-EMF failed to improve flap survival and was associated with molecular changes suggestive of impaired regenerative signaling. Taken together, these findings support the concept of a dose-dependent biological window in which appropriately dosed ELF-EMF may promote tissue repair, whereas excessive exposure may attenuate or negate these beneficial effects.

Random-pattern skin flaps remain fundamental tools in reconstructive surgery, but distal ischemia and necrosis continue to limit their reliability.^7^^,^^8^ Numerous strategies have been investigated to improve flap viability, including ischemic preconditioning, pharmacologic vasodilators, antioxidant agents, hyperbaric oxygen therapy, and growth factor-based approaches.^18^ Although many of these interventions show experimental promise, their clinical application may be limited by cost, invasiveness, logistical complexity, or inconsistent efficacy. ELF-EMF therapy represents an attractive alternative because it is noninvasive, relatively inexpensive, and potentially easy to standardize for clinical use.^2^^,^^5^^,^^6^

The improvement in flap survival observed in the low-dose ELF-EMF group is consistent with prior studies demonstrating favorable effects of electromagnetic stimulation on ischemic tissues and wound healing.^2^, ^3^, ^4^^,^^9^^,^^10^ Previous experimental work has shown that electromagnetic fields can enhance tissue oxygenation, stimulate endothelial and fibroblast activity, and accelerate epithelial regeneration.^2^^,^^3^^,^^11^, ^12^, ^13^ Our results extend these observations by demonstrating that a low-intensity sinusoidal ELF-EMF protocol significantly increased viable flap area compared with both untreated controls and animals exposed to a substantially higher field intensity.

The fact that high-dose ELF-EMF did not improve flap viability strongly supports the concept of a non-linear dose-response relationship. Biological responses to electromagnetic stimulation are thought to occur within discrete “frequency-amplitude windows,” in which certain combinations of field strength and frequency produce beneficial effects while exposures outside these windows are ineffective or potentially detrimental.^5^^,^^6^^,^^35^ The present findings provide additional evidence for this model and underscore the importance of careful dose optimization in future translational studies.

Histopathological findings corroborated the macroscopic observations. The low-dose ELF-EMF group demonstrated accelerated re-epithelialization, increased vascular density, better organized collagen deposition, and reduced inflammatory cell infiltration. These findings suggest that low-dose ELF-EMF supports multiple stages of wound repair, including epithelial regeneration, angiogenesis, extracellular matrix remodeling, and resolution of inflammation.^12^, ^13^, ^14^, ^15^ Improved collagen architecture may reflect enhanced fibroblast function and more efficient matrix maturation, both of which are critical determinants of flap strength and long-term tissue integrity.^14^^,^^15^

The molecular findings should be interpreted cautiously. In the low-dose ELF-EMF group, simultaneous upregulation of both pro-apoptotic genes (BAX, CASP8, CASP9) and the anti-apoptotic gene BCL2 suggests activation of a coordinated stress-response network rather than a simple anti-apoptotic effect.^21^, ^22^, ^23^, ^24^, ^25^, ^26^, ^27^ Controlled apoptosis is a normal component of tissue remodeling, enabling removal of damaged cells while preserving overall tissue homeostasis. Concurrent induction of BCL2 may reflect compensatory survival signaling aimed at limiting excessive cell loss. Therefore, these transcriptional changes likely indicate tightly regulated cellular adaptation rather than unequivocal promotion or inhibition of apoptosis.

TNF-α expression showed an apparently discordant pattern: mRNA levels were elevated in the low-dose ELF-EMF group, whereas immunohistochemical staining demonstrated relatively weak protein immunoreactivity. Several mechanisms may explain this finding, including temporal differences between transcriptional activation and protein accumulation, post-transcriptional regulation, differences in cellular localization, or the semi-quantitative limitations of immunohistochemistry.^16^, ^17^, ^18^, ^19^, ^20^ An early transient increase in TNF-α transcription may be part of a normal reparative response, while sustained protein expression may be suppressed as inflammation resolves. Accordingly, the TNF-α findings should not be interpreted as evidence of persistent pro-inflammatory activity.

In contrast to the low-dose group, high-dose ELF-EMF was associated with marked downregulation of VEGF and several MAPK pathway-related genes, including MAPK1, MAPK3, and MAPK14. Because these signaling pathways play central roles in angiogenesis, proliferation, and cellular stress adaptation, their suppression may contribute to the absence of histological and functional improvement observed with high-intensity exposure.^28^, ^29^, ^30^ The pronounced reduction in VEGF expression is particularly notable, as impaired angiogenic signaling would be expected to limit neovascularization and compromise survival of ischemic flap tissue.

The role of MAPK signaling in electromagnetic field responses is complex. Previous studies suggest that electromagnetic stimulation may influence MAPK pathways primarily through post-translational activation rather than transcriptional regulation alone.^28^^,^^29^ Nevertheless, the transcriptional suppression observed in the high-dose group is consistent with a biologically unfavorable response and may reflect excessive cellular stress. Additional studies examining phosphorylation status of ERK1/2, JNK, and p38 MAPK proteins would provide a more definitive mechanistic understanding.

Although enhancement of microcirculatory function was a conceptual objective of this study, direct perfusion measurements were not performed. Consequently, conclusions regarding microcirculation are inferred indirectly from flap survival, histological vascularization, and angiogenesis-related gene expression. While these surrogate markers strongly suggest improved perfusion-related healing in the low-dose group, future studies incorporating laser Doppler flowmetry, laser speckle contrast imaging, or indocyanine green angiography are needed to directly quantify perfusion changes induced by ELF-EMF.

The discrepancy between molecular and histological endpoints also highlights the dynamic nature of tissue repair. Gene expression changes may occur rapidly and transiently, whereas histological and immunohistochemical findings reflect cumulative tissue responses over time. Because all analyses were performed at a single postoperative time point, the temporal sequence of molecular events could not be characterized. Serial sampling at multiple intervals would provide valuable insight into the kinetics of ELF-EMF-induced biological responses.

From a translational perspective, the results are encouraging. If similar effects can be reproduced in larger animal models and ultimately in humans, low-dose ELF-EMF could emerge as a practical adjunct for improving flap viability in reconstructive surgery. Potential applications include random-pattern flaps, compromised local flaps, and high-risk reconstructive procedures in patients with diabetes, smoking history, or vascular insufficiency. Because the therapy is noninvasive and does not require systemic drug administration, it may offer a favorable safety and cost profile.^2^^,^^6^

Several limitations should be considered when interpreting these findings. First, the sample size was relatively small (n = 7 per group), and no formal a priori power analysis was performed. Although this sample size is comparable to that used in many exploratory animal studies, the possibility of type II error cannot be excluded. Second, direct measurements of tissue perfusion were not obtained. Third, histological and immunohistochemical analyses were semi-quantitative and performed by a single blinded observer, and interobserver reliability was not assessed. Fourth, most gene-expression findings were not validated at the protein level. Fifth, only a single frequency (50 Hz) and two field intensities were tested. Finally, the study represents a proof-of-concept investigation in a rodent model, and the clinical applicability of these results remains to be established.

Despite these limitations, the study has several important strengths. The experimental design included a clearly defined dose-comparison approach, standardized flap surgery, blinded histological evaluation, and integration of macroscopic, histological, immunohistochemical, and molecular outcome measures. The consistency between flap survival and histological findings adds confidence to the principal conclusion that low-dose ELF-EMF improves tissue repair in this model.

In summary, the present study demonstrates that low-dose sinusoidal ELF-EMF significantly enhances survival and histological markers of repair in a rat random-pattern skin flap model, whereas high-dose exposure does not provide benefit and may suppress regenerative pathways. These findings support the existence of a dose-dependent biological window for ELF-EMF therapy and provide a compelling preclinical rationale for further mechanistic investigations and translational studies.

## Conclusion

Low-dose sinusoidal ELF-EMF (0.2 mT, 50 Hz) significantly improved survival and histological markers of tissue repair in a rat random-pattern skin flap model. These beneficial effects were associated with enhanced vascularization, improved collagen organization, and modulation of apoptosis- and inflammation-related gene expression. In contrast, high-dose ELF-EMF did not improve flap viability and was associated with suppression of several regenerative signaling pathways.

Taken together, these findings support the existence of a dose-dependent biological window for ELF-EMF therapy. However, given the exploratory nature of this preclinical study and the absence of direct perfusion measurements and protein-level validation, the results should be interpreted cautiously. Further mechanistic studies and well-designed clinical investigations are necessary before ELF-EMF can be considered for routine use as an adjunct in reconstructive flap surgery.

## Authors’ contributions

Mehmet Goktug Esmer: Conceptualization, Methodology, Investigation, Formal analysis, Data curation, Writing-Original Draft.

Aysun Ozbay Onal: Investigation, Data curation, Formal analysis, Visualization, Writing-Original Draft, Writing-Review & Editing.

Mustafa Onal: Investigation.

Sevda Demir: Investigation, Formal analysis.

Seden Yurtoglu: Investigation, Resources.

Merdan Serin: Supervision, Writing-Review & Editing.

All authors have read and approved the final manuscript.

## Ethics approval

All experimental procedures were approved by the Maltepe University Local Ethics Committee for Animal Experiments (Approval date: February 27, 2025, Protocol No: 2025.02.02).

## Funding

This research received no financial support from any public, commercial, or not-for-profit funding agencies.

## Consent for publication

Not applicable.

## Availability of data and materials

The datasets generated and analyzed during the current study are available from the corresponding author upon reasonable request.

## Data availability

All data supporting the findings of this study are included in this article. Additional raw data are available from the corresponding author upon reasonable request.

## Declaration of competing interest

The authors declare that they have no competing interests.

## References

[1] Hashimoto I., Abe Y., Ishida S., Kashiwagi K., Mineda K., Yamashita Y. (2016). Development of skin flaps for reconstructive surgery : random pattern flap to perforator flap. J Med Investig.

[2] Stojanovic M., Rai V., K Agrawal D. (2024). Effect of electromagnetic field on proliferation and migration of fibroblasts and keratinocytes: implications in wound healing and regeneration. J Biotechnol Biomed.

[3] Costantini E., Sinjari B., D’Angelo C., Murmura G., Reale M., Caputi S. (2019). Human gingival fibroblasts exposed to extremely low-frequency electromagnetic fields: in vitro model of wound-healing improvement. Int J Mol Sci.

[4] Preetam S., Ghosh A., Mishra R., Pandey A., Roy D.S., Rustagi S. (2024). Electrical stimulation: a novel therapeutic strategy to heal biological wounds. RSC Adv.

[5] Krylov V.V., Osipova E.A. (2023). Molecular biological effects of weak low-frequency magnetic fields: frequency–Amplitude efficiency windows and possible mechanisms. Int J Mol Sci.

[6] Gualdi G., Costantini E., Reale M., Amerio P. (2021). Wound repair and extremely low frequency-electromagnetic field: insight from in vitro study and potential clinical application. Int J Mol Sci.

[7] Uzel K. (2024). Lokal flep uygulamalarının prensipleri (Antegrad homodijital nörovasküler ada flebi, V-Y ilerletme flebi ve Z plasti). TOTBİD Derg.

[8] Duymaz A., Karabekmez E., Kesk‹n M., Sütçü M., Tosun Z. Ekstended V-Y Flep ile Büyük Yüz Defektlerinin Rekonstrüksiyonu. N.D.

[9] Lee J.W., Kim J.Y., Lee N.R., Lee Y.H. (2022). Effect of pulsed electromagnetic fields stimulation on ischemic skin model. Electromagn Biol Med.

[10] Weber R.V., Navarro A., Wu J.K., Yu H.L., Strauch B. (2004). Pulsed magnetic fields applied to a transferred arterial loop support the rat groin composite flap. Plast Reconstr Surg.

[11] D’Angelo C., Costantini E., Kamal M.A., Reale M. (2015). Experimental model for ELF-EMF exposure: concern for human health. Saudi J Biol Sci.

[12] Matic M., Lazetic B., Poljacki M., Djuran V., Matic A., Gajinov Z. (2009). Influence of different types of electromagnetic fields on skin reparatory processes in experimental animals. Lasers Med Sci.

[13] Athanasiou A., Karkambounas S., Batistatou A., Lykoudis E., Katsaraki A., Kartsiouni T. (2007). The effect of pulsed electromagnetic fields on secondary skin wound healing: an experimental study. Bioelectromagnetics.

[14] Lhlberg L., Haukipuro K., Risteli L., Oikarinen A., Kairaluoma M.I., Risteli J. (1993). Collagen Synth Intact Skin Is Suppressed Dur Wound Heal.

[15] Callaghan M.J., Chang E.I., Seiser N., Aarabi S., Ghali S., Kinnucan E.R. (2008). Pulsed electromagnetic fields accelerate normal and diabetic wound healing by increasing endogenous FGF-2 release. Plast Reconstr Surg.

[16] Zou J., Chen Y., Qian J., Yang H. (2017). Effect of a low-frequency pulsed electromagnetic field on expression and secretion of IL-1β and TNF-α in nucleus pulposus cells. J Int Med Res.

[17] Goh J., Suh D., Um D.Y., Chae S.A., Park G.S., Song K. (2025). Continuous exposure to 60 hz extremely low frequency magnetic field at 10–14 mT promotes various human cell proliferation by activating extracellular-signal-regulated kinase. Biochem Biophys Res Commun.

[18] Afrooghe A., Damavandi A.R., Ahmadi E., Jafari R.M., Dehpour A.R. (2023). The current state of knowledge on how to improve skin flap survival: a review. J Plast Reconstr Aesthet Surg.

[19] Costantini E., Aielli L., Serra F., De Dominicis L., Falasca K., Di Giovanni P. (2022). Evaluation of cell migration and cytokines expression changes under the radiofrequency electromagnetic field on wound healing In vitro model. Int J Mol Sci.

[20] Toledano-Macías E., Martínez-Pascual M.A., Cecilia-Matilla A., Bermejo-Martínez M., Pérez-González A., Jara R.C. (2024). Radiofrequency currents modulate inflammatory processes in keratinocytes. Int J Mol Sci.

[21] Cichon N., Synowiec E., Miller E., Sliwinski T., Ceremuga M., Saluk-Bijak J. (2020). Effect of rehabilitation with extremely low frequency electromagnetic field on molecular mechanism of apoptosis in post-stroke patients. Brain Sci.

[22] Martinelli I., Cinato M., Keita S., Marsal D., Antoszewski V., Tao J. (2022). Cardiac cell exposure to electromagnetic fields: focus on oxdative stress and apoptosis. Biomedicines.

[23] Wang Y., Wu Y., Wang P., Luo J., Rui Y. (2022). Exploration of the protective mechanism of bax removal against ischemia reperfusion injury of skin flap through the p38 mitogen-activated protein kinase pathway. Oxid Med Cell Longev.

[24] Deng J., Wang K., Yang J., Wang A., Chen G., Ye M. (2024). β-caryophyllene promotes the survival of random skin flaps by upregulating the PI3K/AKT signaling pathway. Phytomedicine.

[25] Ye P., Wu X., Gu R., Zhu H., Chen J., Dai Y. (2025). ACNO hydrogel enhances diabetic wound healing by modulating the bcl-2/bax/caspase-3/PARP pathway. Int Immunopharmacol.

[26] Balci Ç., Özcan M.S., Aşci H., Karabacak P., Kuruşçu O., Taner R. (2025). Radiofrequency electromagnetic and pulsed magnetic fields protected the kidney against lipopolysaccharide-induced acute systemic inflammation, oxidative stress, and apoptosis by regulating the IL-6/HIF1α/eNOS and Bcl2/bax/Cas-9 pathways. Med (Lith).

[27] Shoshan-Barmatz V., Arif T., Shteinfer-Kuzmine A. (2023). Apoptotic proteins with non-apoptotic activity: expression and function in cancer. Apoptosis.

[28] Yumoto H., Hirao K., Tominaga T., Bando N., Takahashi K., Matsuo T. (2015). Electromagnetic wave irradiation promotes osteoblastic cell proliferation and up-regulates growth factors via activation of the ERK1/2 and p38 MAPK pathways. Cell Physiol Biochem.

[29] Ryu Y., Wague A., Liu X., Feeley B.T., Ferguson A.R., Morioka K. (2024). Cellular signaling pathways in the nervous system activated by various mechanical and electromagnetic stimuli. Front Mol Neurosci.

[30] Patruno A., Ferrone A., Costantini E., Franceschelli S., Pesce M., Speranza L. (2018). Extremely low-frequency electromagnetic fields accelerates wound healing modulating MMP-9 and inflammatory cytokines. Cell Prolif.

[31] Tenuzzo B., Vergallo C., Dini L. (2009). Effect of 6 mT static magnetic field on the bcl-2, bax, p53 and hsp70 expression in freshly isolated and in vitro aged human lymphocytes. Tissue Cell.

[32] Mendoza-Mari Y., Rai V., M Radwan M., Brazdzionis J., A Connett D., E Miulli D. (2024). Modulation of inflammatory response by electromagnetic field stimulation in traumatic brain injury in Yucatan swine. J Surg Res (Houst).

[33] Tohidi F.Z., Sadr-Nabavi A., Haghir H., Fardid R., Rafatpanah H., Azimian H. (2021). Long-term exposure to electromagnetic radiation from mobile phones can cause considerable changes in the balance of bax/Bcl2 mRNA expression in the hippocampus of mice. Electromagn Biol Med.

[34] Kim J.H., Yu D.H., Kim H.R. (2017). Activation of autophagy at cerebral cortex and apoptosis at brainstem are differential responses to 835 MHz RF-EMF exposure. Korean J Physiol Pharmacol.

[35] Zhou J., Gao Y.H., Zhu B.Y., He W.F., Wang G., Xian C.J. (2021). The frequency window effect of sinusoidal electromagnetic fields in promoting osteogenic differentiation and bone formation involves extension of osteoblastic primary cilia and activation of protein kinase A. Cell Biol Int.

[36] Shen Y., Xia R., Jiang H., Chen Y., Hong L., Yu Y. (2016). Exposure to 50Hz-sinusoidal electromagnetic field induces DNA damage-independent autophagy. Int J Biochem Cell Biol.

[37] Kim S.J., Jang Y.W., Hyung K.E., Lee D.K., Hyun K.H., Jeong S.H. (2017). Extremely low-frequency electromagnetic field exposure enhances inflammatory response and inhibits effect of antioxidant in RAW 264.7 cells. Bioelectromagnetics.

